# Combining Measures of Signal Complexity and Machine Learning for Time Series Analyis: A Review

**DOI:** 10.3390/e23121672

**Published:** 2021-12-13

**Authors:** Sebastian Raubitzek, Thomas Neubauer

**Affiliations:** Information and Software Engineering Group, Institute of Information Systems Engineering, Faculty of Informatics, TU Wien, Favoritenstrasse 9-11/194, 1040 Vienna, Austria; thomas.neubauer@tuwien.ac.at

**Keywords:** hurst exponent, chaos, Lyapunov exponents, neural networks, time series prediction, deep learning, machine learning, LSTM, R/S analysis, regression analysis, time series data

## Abstract

Measures of signal complexity, such as the *Hurst exponent*, the *fractal dimension*, and the *Spectrum of Lyapunov exponents*, are used in time series analysis to give estimates on persistency, anti-persistency, fluctuations and predictability of the data under study. They have proven beneficial when doing time series prediction using machine and deep learning and tell what features may be relevant for predicting time-series and establishing complexity features. Further, the performance of machine learning approaches can be improved, taking into account the complexity of the data under study, e.g., adapting the employed algorithm to the inherent long-term memory of the data. In this article, we provide a review of complexity and entropy measures in combination with machine learning approaches. We give a comprehensive review of relevant publications, suggesting the use of fractal or complexity-measure concepts to improve existing machine or deep learning approaches. Additionally, we evaluate applications of these concepts and examine if they can be helpful in predicting and analyzing time series using machine and deep learning. Finally, we give a list of a total of six ways to combine machine learning and measures of signal complexity as found in the literature.

## 1. Introduction

The rise of artificial intelligence resulted in increased use of machine and deep learning for analysis and predictions based on historical data instead of employing mechanistic expert models. The outcomes of these predictions are encouraging, e.g., in solid-state physics, first-principle calculations can be sped up [1], or solar radiation can be predicted using machine learning methods [2]. In biology, machine learning can be applied, e.g., to genomics, proteomics, and evolution [3]. In medicine, researchers used machine learning to improve diagnosis using collected information from the past [4]. In finance, the applications range from risk management or the construction of portfolios to designing and pricing securities [5]. In agriculture, machine learning can be used to predict yields and give estimates on the nitrogen status [6].

However, while these approaches provide promising results in some research areas, the results are inferior in some others, depending on the available data and the system’s complexity. Though it can not be generalized what sort of data is necessary to perform machine learning successfully, the two main reasons, assuming one has optimized the algorithm for the regarded task, for machine learning to fail are:

(i) Lack of data, meaning that the overall amount of data is not enough to train a model properly.
(ii) Lack of good data, meaning that despite there is a sufficient amount of data available, the inherent information is not enough to achieve good results.

One way to improve machine learning approaches suffering from these problems is by studying complex systems, i.e., chaos theory. The reason for this is that real-life systems are highly complex and non-linear systems. Therefore, complexity analysis and ideas from chaos theory should be considered when analyzing or predicting real-life systems. Therefore, data-driven analysis and prediction methods can be improved, taking into account the complexity and non-linear behavior of the system under study. e.g., in the case of a general lack of data one can employ interpolation techniques such as fractal interpolation [7], or stochastic interpolation approaches [8], to increase the overall amount of data. The advantage of these interpolation techniques, in contrast to, e.g., linear or polynomial interpolation, is that the interpolated data can be tailored to match the complexity of the original data.

The study of complex systems had its peak in the 20th century, and many techniques have been developed to characterize and analyze chaotic systems, i.e., complexity measures. Furthermore, chaos theory has a widespread spectrum of applications ranging from medicine [9], finance [10] to physics [11], to name a few applications. Though chaos theory is not that popular among computer scientists, there is significant potential for future applications. For example, data-driven analysis/prediction approaches in agriculture could greatly benefit from considering the complexity of historical data. In agricultural systems, turbulence and complex non-linear behavior can be observed for many phenomenons affecting the overall system [12], such as weather [13], irrigation [14] or environmental pollution [15].

Though there are numerous other applications of measures of signal complexity, only a few publications exist which combine artificial intelligence and measures of signal complexity for time series analysis. When referring to complexity measures or measures of signal complexity, we mean all sorts of entropy, information, order-, disorder-measures one can perform on time series data. This article is a survey of those applications.

For this purpose, we searched *google scholar* for combinations of words synonymous with machine and deep learning applications. Those words, together with words synonymous for the study of complex systems, yielded the list of publications reviewed in Section 3. The result is a list of 18 publications that combine machine learning and complexity measures for real-life time series analysis in one way or another. E.g., in [16], a hybrid approach of fuzzy logic, *fractal dimension*, and a neural network are used to make predictions of time series. In [17], several indices of emerging economics are examined using the *Hurst exponent* and the *fractal dimension*, thus indicating a long term memory in the time series data, and afterward are forecast using machine learning methods.

The reason for this relatively low number of 18 combined applications of machine learning and chaos theory for time series analysis is most likely to be found in the education of computer scientists. Most modern computer scientists are trained in math and physics, but the study of complex systems (chaos theory) is, in most cases, not part of the curriculum. Therefore this survey aims to get computer scientists working with artificial intelligence in touch with ideas from the study of complex systems to boost their research. Consequently, we want to find out what combinations have already been successfully implemented in combination with machine learning, where one approach can benefit from the other, and how the combined approaches can be categorized. We focus on the discussed complexity measures and only briefly discuss the corresponding machine learning algorithms.

Finally, we highlight possible contributions of a combined approach:

- One may save computational resources due to a clever feature selection based on a data’s inherent complexity.
- Machine learning results can be checked not only on their accuracy but also on the algorithms capability to reconstruct the characteristics of the original data, thus providing a more profound check for generalization of a trained model.
- Additional complexity-based features can improve the accuracy of the model.
- The complexity of the data under study can be used to alter and improve the architecture of the employed algorithm, e.g., adapt the number of input nodes of a neural network to the long-term memory of the data.
- Complexity measures provide additional arguments for analysis, e.g., why is an algorithm not capable of long-term predictions on a specific data set.
- Complexity measures provide a tool to identify regions of predictability and/or non-predictability within a data set. E.g., given financial data, the inherent complexity may be used to identify volatile non-predictable periods.
- Complexity measures can be used as a filter for predictions to improve machine learning ensemble approaches [18].

This article is structured as follows:

In Section 2, we explain the employed approach to search and find relevant publications. Further, we explain how we extracted and concluded the information from all listed publications. Section 3 gives the list of all publications that were used for this research and briefly summarizes the approach taken. We provide all complexity measures used in the featured publications in Section 4 and refer to where they were used. Section 5 lists all machine learning approaches and links them to the publications and the used complexity measures. We finally conclude our findings in Section 6 and sort all publications into six types of approaches for combining machine learning and complexity measures for time series data. Further, we give some ideas on future combinations and applications of machine learning and complexity measures. We also added a table summing up our findings, which can be found in Appendix B.

## 2. Methodology

For this review we performed an online search on google scholar. We searched for the following key words:

The following two lists give the range of search strings which we combined to yield the list of publications from Section 3.

Search strings to indicate machine learning/artificial intelligence:

- Machine Learning
- Artificial Intelligence
- Neural Network
- RNN *or* Recurrent Neural Network
- LSTM *or* Long Short Term Memory
- ANN *or* Artifical Neural Network
- SVM *or* Support Vector Machines

Search strings to indicate methods from chaos theory:

- Chaos
- Hurst Exponent
- Chaotic
- Fractal
- Fractal Dimension
- Lyapunov Exponent
- Entropy

Further, we added the words time series, forecast, and prediction to target time series data applications specifically.

### Exclusion Criteria

The previously mentioned search strings (or the combined search strings) yielded many publications which had to be filtered. Thus, we used the following criteria to guarantee a focused review process. We excluded all publications that:

- Did not specifically deal with time-series data.
  This is necessary as most measures of signal complexity, e.g., the Hurst exponent, the spectrum of Lyapunov exponents, etc., deal with time-series data only.
- Mentioned, but did not employ whether machine learning techniques or ideas from chaos theory and/or complexity measures. i.e., no application was shown.
- Deal only with theoretical implications of a combination of the mentioned techniques, i.e., without an actual application and results.
- Included time series classification tasks, e.g., a time series data was classified as a certain behavior.
- Aimed to calculate the complexity of a signal using a machine learning or neural network approach.
- Analyzed only model data or artificial data.
- Included phase space reconstruction approaches, such as methods to find a time series’s embedding dimension or time delay. (Note that for some complexity measures it is necessary to find a suitable phase space embedding, thus an embedding dimension and a suitable time delay. However, as we focus on the complexity measures, we did not include articles that only did a phase space reconstruction.)

Section 3 shows the remaining results that we chose for this review.

We next analyzed all used complexity measures and machine learning algorithms. We briefly explain the basic idea, and in terms of the complexity measures, describe how they are used in combination with machine learning approaches. Here our focus is on explaining the employed complexity measures and not the machine learning approaches. Still, we briefly discuss the employed machine learning approaches but refer to the mentioned sources for each algorithm for an in-depth treatment of the subject. Thus, we sort all featured publications by the employed complexity measures, their machine learning approaches, and lastly, according to their approach on combining complexity measures and machine learning. Finally, we discuss some future ideas.

## 3. Relevant Publications

There are several applications of complexity measures to machine learning. Here we give an overview of applications, results and discuss the applied techniques. For this reason 18 publications, combining machine learning and chaos theory are listed.

1. Ref [19]: A back-propagation neural network is used to capture the relationship between technical indicators and of the index in the market under study over time. Using the neural network, one can achieve better results than conventional ARIMA(Auto-Regressive Integrated Moving Average) models. *R/S analysis* is used to identify if Kuala Lumpur Composite Index (KLCI) is a random process or if it features long-term memory. A *Hurst exponent* indicates a long memory effect. Afterward, the time series is scrambled, and a new *Hurst exponent* calculated, which is significantly lower. This supports the thesis for KLCI to have a long-term memory.
2. Ref [16]: This research analyzes U.S./Mexico exchange rates for their predictability using different methodologies. The time series is clustered into several pieces. For each piece, the *fractal dimension* is calculated. Then fuzzy membership functions for *fractal dimension*, position, and the geometry of the sub-series are defined. Based on this, predictions are made using fractal-enhanced fuzzy logic and compared to neural network predictions. Here neural networks seem to work better on short-term predictions, but the fuzzy logic approach performs better in the long term.
3. Ref [20]: A layered back-propagation artificial neural network model with feedback connection was used for the study of the solar wind magnetosphere coupling and prediction of the geomagnetic $D_{st}$ index. The *local Hölder exponent*, α, was used to analyze local scaling and singularity properties. The multifractality of the observed fluctuations led to α changing from point to point. The local Hölder exponent was added to the input layer of the neural network resulting in superior performance compared to an approach without the *local Hölder exponent*.
4. Ref [21]: The Nikkei stock prices over 1500 days are analyzed and predicted using fractal analysis, i.e., *Hurst exponent*, *fractal dimension*, and auto-correlation coefficient. The *fractal dimension* and the *Hurst exponent* show that the discussed time series follows a persistent behavior and can, therefore, be forecast theoretically. The fractal analysis also showed that data for three days provided the strongest correlation and, therefore, declared 3 and 5 input nodes. The results showed that the error is significantly lower for three input nodes than for five, and the training could be done using fewer training epochs.
5. Ref [22]: Here, it is stated that time series with a larger *Hurst exponent* can be predicted more accurately by back-propagation Neural Networks than those with *H* values close to 0.5. The *Hurst exponent* for all 1024 trading day periods of the Dow-Jones index from 2 January 1930, to 14 May 2004, is calculated and analyzed. Periods with large *Hurst exponent* can be forecast with higher accuracy than those with low accuracy. The results suggest that the *Hurst exponent* can be used to guide data selection before forecasting. Furthermore, the embedding dimension of the time series under study and the corresponding time delay calculated via minimal mutual information were used to set the input nodes for the neural networks, i.e., The embedding dimension defined the number of input nodes, and the time delay defined the separation between the time steps fed into the neural network. This idea is based on Taken’s theorem for phase space reconstruction.
6. Ref [23]: Stock market closing prices were predicted using an enhanced evolutionary artificial neural network model (EANN). *R/S analysis* was used to calculate the *Hurst exponent* for different scales in the range of $\left[3,50\right]$ for each time series. The reason for doing this for different sizes of subsets is that one identifies the length of maximal persistency, i.e., the size of subsets where the averaged *Hurst exponent* is maximal. The experiment shows that when finding the scale with the maximal *Hurst exponent*, deviations from that scale shift *H* towards 0.5, meaning that information on persistency in the time series diminishes when observing other scales. Though those *Hurst-exponent-based* models did not outperform the regular EANN models, when considering trading strategies using the *Hurst-exponent-based models*, the average returns are higher than for other models. One interpretation is that the Hurst-based models capture essential information about upward/downward trends in the time series.
7. Ref [24]: According to the efficient market hypothesis, stock prices follow a random walk pattern and can not be predicted with high accuracy. In this research, the Dow Jones Industrial Average is analyzed for its predictability and complexity. Using the *Hurst exponent*, one can identify if a time series is a random walk process or features long-term memory. Here the *Hurst exponent* is used to identify regions in a time series that have non-random characteristics, i.e., H≠0,5. In these regions, predictions are made using artificial neural networks, decision trees, and k-nearest neighbor approaches. Using these techniques and ensembles, accuracy up to 65% was achieved. Further, the embedding dimension and the time delay of the data under study were calculated to alter the architecture of the algorithm, i.e., adapt the input to the characteristics of the supposed underlying complex system.
8. Ref [25]: Many different chaotic time-series predictions are made using a NARX (Nonlinear Autoregressive model process with eXogenous input) dynamic recurrent neural network. The analyzed time series are Chaotic Mackey-Glass time series, Fractal Weierstrass time series, and BET time series (average of daily closed prices for nine representatives, most liquid listed companies at the Bucharest Stock Market). The *Hurst exponent* with H≠0,5 suggests that all three time-series are predictable. For the first two, NARX neural networks perform very well, but for the third, despite having a high *Hurst exponent*, it worked not as well as for the others. The reason for this is that the third is the only real-life time series featured in the selection.
9. Ref [26]: The *fractal dimension* of data sets is used to select features to make predictions with. Afterward, Support Vector Machines (SVM) are used to make predictions. A total of 19 features is used to forecast The Shanghai Stock Exchange Composite Index (SSECI). Using fractal feature selection, this number is reduced to distinguish between "noise" and relevant data to predict future behavior. The results show that higher accuracy is achieved when fractal feature selection is applied. Further, fractal feature selection outperforms five other feature selection methods.
10. Ref [27]: Different AI methodologies, i.e., random forest, neural networks, and Fuzzy Inductive Reasoning (FIR), are proposed to perform short-term electric load forecasting (24h). A feature selection process based on *Shannon entropy* is used to reduce the number of features fed into the learning algorithms. FIR outperformed the random forest and neural network approaches.
11. Ref [28]: Several stock indices of developed and emerging markets are examined if they sport a long-term memory or follow a more random behavior. Rescaled range analysis is performed to calculate the *Hurst exponent* and the *fractal dimension* of those markets. The fractal analysis shows that those markets show persistence and can, therefore, theoretically be forecast. Afterward, machine/deep learning methods, i.e., adaptive neuro-fuzzy inference system, dynamic evolving neuro-fuzzy inference system, Jordan neural network, support vector regression, and random forest, are used to forecast further market development. The results show that these data sets can effectively be forecast, and random forest performed best in this study.
12. Ref [17]: A framework for forecasting exchange rates is presented. Different exchange rates are investigated using R/S analysis, the Hurst exponent, and the fractal dimension of time series, thus showing persistent behavior in the considered time series data. Afterward, the time series data are decomposed into wavelets and predicted using Random Forest and a bagging-based decision tree algorithm. The results show that both approaches performed well for the task at hand.
13. Ref [29]: The scope of this paper is to verify the existence of a relationship between long-term memory in fractal time series and the accuracy of neural networks to forecast them. Brazilian financial assets traded at BM&FBovespa, specifically public companies shares and real estate investment funds, were considered, and their long-term memory was estimated using *R/S analysis* and the *Hurst exponent*. Time lagged feedforward neural networks with back-propagation supervised learning process and gradient descent for error minimization were used to predict future prices. The study shows that one can achieve higher returns when considering time series with higher *Hurst exponent* and leaving out anti-persistent time series, i.e., data with a *Hurst exponent* H<0.5.
14. Ref [30]: This research aims to predict stock market data, i.e., seven different recognized indexes, using three different neural network architectures, i.e., a feed-forward neural network, a cascade forward neural network, and learning vector quantization. Further, wavelet entropy and the Hurst exponent of the data under study are calculated for analysis and as an additional feature to be fed into the algorithms. The results show that the additional features improved the accuracy of the predictions.
15. Ref [31]: Eight different stock indices were analyzed and predicted using *Hurst exponent*, *Shannon entropy*, and *Rényi entropy*. Furthermore, the data was augmented using linear interpolation to generate more data points between actual data points. The final data set consists of eight stock indices, where for each of the indices, the features Open, High, Low, Close, *Hurst exponent*, *Shannon entropy*, Rényi entropy were considered. This generated an overall data set with 56 features. Then the whole dataset was normalized using the min-max normalization method, thus resulting in a total of 150 features. Predictions were made using Multi-Layer Regression (MLR), Support Vector Regression (SVR), and Feed Forward Back Propagation models. All models were tested using a different assemble of *Hurst exponent*, *Rényi entropy*, *Shannon entropy* components. The results show that the *Hurst exponent* component is crucial for making good predictions. The best results were obtained when using Feed Forward Back Propagation and including all three complexity features, i.e., *Hurst exponent*, *Rényi entropy*, *Shannon entropy*.
16. Ref [32]: Here, different approaches to predict the direction of US stock prices are compared with each other. Several algorithms were employed for this task, i.e., logistic regression, a multilayer perceptron (MLP), random forest, XGBoost, and a long short term memory (LSTM) recurrent neural network. Furthermore, effective transfer entropy (ETE) is calculated for analysis and as an additional feature to be fed into the algorithms. The results show that the predictions could be improved by adding ETE to the input and, second, that LSTM and the MLP perform best for this task.
17. Ref [7]: A fractal interpolation method is compared to a linear interpolation method to improve neural network time-series predictions. The fractal interpolation method uses the Hurst exponent to match the complexities of given sub-intervals of the time series data under study. Further, the neural network architecture is a Long Short Term Memory (LSTM) recurrent neural network. Furthermore, all results and data are analyzed using the Hurst exponent, the fractal dimension, and the spectrum of Lyapunov exponents. The results show that neural network predictions can significantly be improved using both linear and fractal interpolation techniques.
18. Ref [33]: The fractal interpolation method from [7] is used on a total of five datasets, with varying numbers of interpolation points. Further, these five data sets are then forecast using randomly parameterized ensembles of LSTM neural networks. These randomly parameterized ensemble predictions are then filtered using different complexity measures: The Hurst exponent, the spectrum of Lyapunov exponents, Fisher’s information, SVD entropy and Shannon’s entropy. The predictions could be improved by filtering the ensemble predictions. Further, the predictions outperformed baseline predictions using LSTM, GRU and RNN neural network approaches with one hidden layer. Further, all interpolated data sets are also analyzed using the previously mentioned complexity measures.

## 4. Complexity Measures

This section lists all complexity measures used in the discussed publications (Section 3), gives additional references and mentions how the complexity measures are used in combination with machine learning.

A table briefly summarizing and comparing the featured complexity measures can be found in Appendix A.

### 4.1. The Hurst Exponent

The rescaled range analysis (*R/S analysis*), as invented by Harold Edwin Hurst [34], is the procedure of determining the Hurst exponent “*H*” of time series data. The Hurst exponent is a measure of the long-term memory of a time series or a process.

As we only outline the main aspects of R/S analysis, we refer to [34,35] for a detailed discussion of the topic.

For a given a signal $\left[x_{1},x_{2},\ldots ,x_{n}\right]$, we find the the average over a period τ (a sub-interval of the signal 1≤τ≤n) as:

(1) $${\langle x\rangle }_{\tau ,k}\;=\;\frac{1}{\tau }\sum_{j=k}^{k+\tau }\;x_{j}\;,$$

where 1≤k≤n for all elements *i* in this interval such that k≤i≤k+τ.

We further find an accumulated departure $\delta x\left(i,\tau ,k\right)$ over period a period i=1,2,…,τ is:

(2) $$\delta x\left(i,\tau ,k\right)\;=\;\sum_{j=k}^{i}\left(x_{j}-{\langle x\rangle }_{\tau ,k}\right)\;$$

The difference between maximal and minimal values of all $x_{i}$ in the interval $\left[k,k+\tau \right]$, i.e., the range *R* of this interval τ is:

(3) $$\begin{array}{cc} R\left(\tau ,k\right)\; & =\;\max\left[\delta x\left(i,\tau ,k\right)\right]\;-\;\min\left[\delta x\left(i,\tau ,k\right)\right]\;,\\ \; & \mathrm{satisfying}\;\;k\leq i\leq k+\tau \;. \end{array}$$

The standard deviation for each sub interval is given as:

(4) $$S\left(\tau ,k\right)\;=\;\sqrt{\frac{1}{\tau }\sum_{i=k}^{k+\tau }{\left[x_{i}-{\langle x\rangle }_{\tau ,k}\right]}^{2}}\;.$$

Averaging the range and the standard deviation over all possible *k* yields:

(5) $$\begin{array}{cc} \; & R\left(\tau \right)\;=\;\frac{\sum_{k}R\left(\tau ,k\right)}{n_{k}}\\ \; & \;\mathrm{and}\\ \; & S\left(\tau \right)\;=\;\frac{\sum_{k}S\left(\tau ,k\right)}{n_{k}}\;, \end{array}$$

where 1≤k≤n, k≤i≤k+τ and $n_{k}$ is the number of different *k*s. The *Hurst exponent* *H* is then defined using the scaling properties as:

(6) $$\frac{R\left(\tau \right)}{S\left(\tau \right)}\;\propto \;\tau^{H}\;,$$

The asymptotic behavior [34,36], for any independent random process with finite variance is then given as:

(7) $$\frac{R\left(\tau \right)}{S\left(\tau \right)}\;=\;{\left(\frac{\pi }{2v}\tau \right)}^{\frac{1}{2}}\;,$$

thus implies $H=\frac{1}{2}$. However, this is only true for completely random processes. For real-life data, we expect $H\neq \frac{1}{2}$, as real-life processes usually feature long-term correlations.

The value of *H* varies as 0<H<1. A value of H<0.5 indicates anti-persistency, i.e., low values follow on high values and vice versa, thus heavily fluctuating, but not completely random. Values close to 0 indicate very strong anti-persistency. Contrarily, for values of H>0.5 we expect persistent behavior and very strong persistency for values close to 1.

When it comes to combined applications with machine learning and R/S Analysis/the Hurst exponent, we find the following publications:

Ref [19]: In this research, the Hurst exponent was used to show long term memory in time series data as an argument for its predictability when using artificial neural networks for stock market data prediction.

Ref [21]: Here, the Hurst exponent was used to estimate the predictability of stock market data, and later to tailor the input nodes of a neural network architecture.

Ref [23]: For this study on stock market data predictability, the Hurst exponent was used to set the input window of an evolutionary neural network architecture.

Ref [22]: Here, it was shown that time-series data with a larger Hurst exponent can be predicted more accurately using an artificial neural network than data with a Hurst exponent closer to 0.5.

Ref [24]: In this research, the Hurst exponent was used to identify regions in stock market data with higher predictability for several different machine learning approaches.

Ref [25]: Here, the Hurst exponent is used as a measure for the predictability of machine learning approaches.

Ref [17]: The researchers used the Hurst exponent to reject the random walk hypothesis of stock market data and as a measure for predictability.

Ref [28]: The researchers used the Hurst exponent to reject the random walk hypothesis of stock market data and as a measure for predictability.

Ref [29]: Here, the Hurst exponent was used to identify time series or regions with higher predictability to improve forecasts.

Ref [31]: Here, the Hurst exponent is used as an additional feature in the input of the employed algorithms to improve forecasts.

Ref [30]: Here, the Hurst exponent is used as an additional feature in the input of the employed algorithms to improve forecasts.

Ref [7]: Here, the Hurst exponent is used to, first, match a fractal interpolation to a data set and second, to analyze all data under study.

The Hurst exponent is used in [33] to filter LSTM ensemble predictions and to discuss all data under study.

### 4.2. Fractal Dimension of a Time-Series

The *fractal dimension* of a time series measures the complexity of a signal. The basic idea is can be understood as the area inhabited by the time series on a two-dimensional plane, i.e., the time series being placed upon a grid of equal spacing and checking the number of grid boxes necessary for covering it. Therefore, the *fractal dimension* is the ratio of the boxes covering the time series and the overall area of the plot. This process is referred to as box-counting. It has also been characterized as a measure of the space-filling capacity of a time series that tells how a fractal scales differently from the space it is embedded in; a *fractal dimension* does not have to be an integer. The *fractal dimension* of a self-affine time series can have values 1<D<2.

Additionally, the *fractal dimension* is closely related to the *Hurst exponent* [21,37,38,39]. Therefore *R/S analysis* can be employed to calculate the *fractal dimension* of a time series using:

(8) D=2−H.

This originates from the fact that the long-range dependence (∼H) is closely related to self-similarity (∼D), and therefore the variability of the process.

Other ways to calculate the fractal dimension are the algorithm by Katz [40]; the algorithm by Higuchi [41]; and the algorithm by [42].

In [16], the fractal dimension is used as an additional feature for a fuzzy-logic-based forecasting technique, i.e., the fractal dimension is included when making the rules. Furthermore, a novel approach to calculate the fractal dimension of a geometric object, based on fuzzy logic is presented.

Ref [21]: This research employs the fractal dimension, alongside the Hurst exponent, of the time series under study to identify the best number of input nodes for a neural network. Further, the fractal dimension and the Hurst exponent are used to show that the time series under study is a non-random process that can in-theory be predicted.

In [26], the fractal dimension is used, together with an ant colony algorithm, as a tool for feature selection in a stock trend time series prediction task.

In [28], the fractal dimension, alongside the Hurst exponent, is used to indicate predictive behavior instead of a purely random behavior of the considered stock market data. Furthermore, *Joseph’s effect* and *Noah’s effect* are considered and discussed [43].

Ref [17]: Just as above, the fractal dimension, alongside the Hurst exponent, is used to show that the considered stock market data can in-theory be predicted. Furthermore, *Joseph’s effect* and *Noah’s effect* are considered and discussed [43].

Ref [7]: The fractal dimension alongside other complexity measures is used to analyze the data under study and the presented fractal interpolated data and how this relates to a neural network time series approach.

### 4.3. The Local Hölder Exponent

Following [20,44,45]:

Given a signal $x_{i}=\left[x_{1},x_{2},\ldots ,x_{n}\right]$, we find the corresponding amplitudes as:

(9) $${\hat{x}}_{i}=\frac{x_{i}}{\sqrt{\sum_{i=1}^{n}x_{i}^{2}}}\;\mathrm{such}\mathrm{that}:\sum_{i=1}^{n}{\hat{x}}_{i}^{2}=1\;.$$

Next, very similar to R/S analysis, find windows or periods as $\left(i-\tau ,i\right)$ and the corresponding signal energies as:

(10) $$E_{i,\tau }=\sum_{i-\tau }^{i}{\hat{x}}_{i}^{2}\;.$$

Next we find the local Hölder or singularity exponent as:

(11) $$\alpha_{i}\left(\tau \right)=\frac{\log\left(E_{i,\tau }\right)}{\log\left(\tau \right)}\;.$$

Similar to the Hurst exponent or Fractal dimension, the exponent α quantifies degree of regularity or irregularity (singularity) in a distribution or a function at a point *i* for different possible window lengths τ, i.e., calculated via regression analysis.

Furthermore, for monofractal process, e.g., fractional Brownian motion, $alpha_{i}$ will be constant for all *i*, where for the case of multi-fractal processes, alpha changes from point to point, thus also provides a measure for the fractal behavior of time series data.

Ref [20]: In this research the local Hölder exponent was used, first o analyze the data under study, and second as an additional input feature to improve predictions.

### 4.4. Transfer Entropy *(TE)* and Effective Transfer Entropy *(ETE)*

Following [32,46,47]:

TE is used to measure the Granger-causal relationship between two processes, thus it can detect a non-linear Granger-causal relationship and is used in various disciplines, such as neuroscience and finance.

For two interacting time series *X* and *Y*, i.e., a variable *Y* can affect the future time point of the variable *X*. Then, if we consider the time series *X* to be Markov process of degree *k*, the state $x_{i+1}$ is thus affected by *k* previous states of the same variable:

(12) $$p\left(x_{n+1}\left|\;x_{n},x_{n-1},\ldots ,x_{0}\right.\right)=p\left(x_{n+1}\left|\;x_{n},x_{n-1},\ldots ,x_{n-k+1}\right.\right),\;x_{i}\in X.$$

Here $p\left(A\left|\;B\right.\right)$ is a conditional probability of *A* given *B*, thus $p\left(A,B\right)/p\left(B\right)$. Further, the state $x_{n+1}$ depends on *l* previous states of *Y*, then we define the TE from a variable *Y* to a variable *X* as the average information included in *Y* excluding the information from the past state of *X* to the next state of *X*. Thus, employing Shannon’s entropy concept (see Section 4.6), we can define the TE from *Y* to *X*, where the state $x_{n+1}$ is affected by *k* previous states of *X* and *l* previous states of *Y*, as:

(13) $$\begin{array}{cc} {\mathrm{TE}}_{Y\to X}^{\left(k,l\right)} & =\sum_{i}p\left(x_{n+1},x_{n}^{\left(k\right)},y_{n}^{\left(l\right)}\right)\log_{2}p\left(x_{n+1}\left|\;x_{n}^{\left(k\right)},y_{n}^{\left(l\right)}\right.\right)-\sum_{i}p\left(x_{n+1},x_{n}^{\left(k\right)},y_{n}^{\left(l\right)}\right)\log_{2}p\left(x_{n+1}\left|\;x_{n}^{\left(k\right)}\right.\right)\\ \; & =\sum_{i}p\left(x_{n+1},x_{n}^{\left(k\right)},y_{n}^{\left(l\right)}\right)\log_{2}\frac{p\left(x_{n+1}\left|\;x_{n}^{\left(k\right)},y_{n}^{\left(l\right)}\right.\right)}{\left(x_{n+1}\left|\;x_{n}^{\left(k\right)}\right.\right)}\;. \end{array}$$

Here the index *i* refers to $i=\{x_{n+1},x_{n}^{\left(k\right)},y_{n}^{\left(l\right)}\}$, whereas $x_{n}^{\left(k\right)}=\left(x_{n},x_{n-1},\ldots ,x_{n-k+1}\right)$ and $y_{n}^{\left(l\right)}=\left(y_{n},y_{n-1},\ldots ,y_{n-l+1}\right)$ and $p\left(A,B\right)$ is the joint probability of *A* and *B*.

Setting k=l=1 to express the weak form of the efficient market hypothesis (when applied to financial data), i.e., the current price reflects all past information, we find TE as:

(14) $$\begin{array}{cc} {\mathrm{TE}}_{Y\to X}= & \sum_{i}p\left(x_{n+1},x_{n},y_{n}\right)\log_{2}\frac{p\left(x_{n+1}\left|\;x_{n},y_{n}\right.\right)}{p\left(x_{n+1}\left|\;x_{n}\right.\right)}\\ = & \sum_{i}p\left(x_{n+1},x_{n},y_{n}\right)\log_{2}\frac{p\left(x_{n+1},x_{n},y_{n}\right)p\left(x_{n}\right)}{p\left(x_{n+1},x_{n}\right)p\left(x_{n},y_{n}\right)}\; \end{array}$$

and again $i=\{x_{n+1},x_{n},y_{n}\}$.

Summed up, we can interpret ${\mathrm{TE}}_{Y\to X}$ as the difference between the information for future values of *X* obtained from *X* and *Y* and the information for future values of *X* obtained only from *X*. Thus, a positive value of ${\mathrm{TE}}_{Y\to X}$ indicates that *Y* affects future values of *X*; also, the larger the value, the more significant the information flow. Furthermore, because of its asymmetric properties, we can view ${\mathrm{TE}}_{Y\to X}$ as the in-flow information from *Y* to *X*, and contrary to that, ${\mathrm{TE}}_{X\to Y}$ as the out-flow information from *X* to *Y*.

#### Effective Transfer Entropy

TE measures a statistical dependency between two signals *X* and *Y* regardless of the data type. For TE to be calculated, a large amount of data is necessary. Another disadvantage is that TE includes noise from sample effects or the non-stationarity of the data set under study. To solve the noise issue, one can employ Effective Transfer entropy (ETE) [47]. To calculate ETE, one must first shuffle the elements of each time series understudy to keep the inherent probability distribution but break any causal relationships and dependencies. Then one obtains the TE from these randomized time series data, thus referred to as Randomized Transfer Entropy (RTE). The final step to determine ETE is then to subtract RTE from the original TE.

(15) $${\mathrm{ETE}}_{Y\to X}={\mathrm{TE}}_{Y\to X}-{\mathrm{RTE}}_{Y\to X}\;.$$

Here it is recommended to perform this process, starting with generating randomized time series data several times and average all obtained ETEs.

In [32], ETE is used to enhance the prediction of the direction of US stocks using a variety of different algorithms/approaches. Here it is used as an additional feature in the input of the algorithm.

### 4.5. Rényi Entropy

Following [48]: *Rényi entropy* is one of the families of functionals for quantifying diversity, uncertainty and randomness of a system. The *Rényi entropy* of order *q* is defined for q≥1 (for q→1 as a limit) by:

(16) $$S_{q}\left(X\right)\;=\;\frac{1}{1-q}\;\log_{b}\sum_{i}^{m}p_{i}^{q}\;,$$

where *X* is a discrete random variable, $p_{i}$ is the probability of the event $\left\{X\;=\;x_{i}\right\}$, and *b* is the base of the logarithm. If the probabilities are all the same then all the *Rényi entropies* of the distribution are equal, with $S_{q}\left(X\right)\;=\;\log_{b}m$. In any other case, the entropies are weakly decreasing as a function of *q*. Higher values of *q*, approaching infinity, give the *Rényi entropy* which is increasingly determined by consideration of only the highest probability events. Lower values of *q*, approaching zero, give the *Rényi entropy* which increasingly weights all possible events more equally, regardless of their probabilities.

Rényi entropy, and its alterations (different values of *q*), is one of the families of functionals to quantify the diversity, uncertainty, or randomness of a given system. Ref [31] uses Rényi entropy as an additional feature as input for the employed algorithms to improve predictions.

#### Related Complexity Measures

Taking into account the formal framework given above one can then find further generalizations and/or special cases [48]:

- Hartley entropy or max-entropy:
  The Hartley or max-entropy is Rényi for q=0
- Shannon’s entropy:
  Shannon’s entropy is Rényi entropy with q=1. See Section 4.6.
- Collision entropy:
  Rényi entropy with q=2 is called collision entropy.
- Min-entropy:
  In the limit q→∞, the *Rényi entropy* converges to the *min-entropy* [49].
- Kolomogorov-Sinai entropy:
  Another closely related entropy measure is the *Kolomogorov-Sinai entropy*. Here $x\left(t\right)$ is the trajectory of the dynamic system in an *n*-dimensional phase space with time intervals Δt, t>0. According to [48] the generalized *Kolmogorov-Sinai entropy*$K_{q}$ is then defined as:
  (17) $$K_{q}\left(x_{i}\right)\;=\;-\lim_{r\to 0}\lim_{\Delta t\to 0}\lim_{N\to \infty }\frac{1}{N\;\Delta t}\frac{1}{q-1}\log_{b}\sum_{i_{1},i_{2},\ldots ,i_{N}}^{m\left(r\right)}p_{i_{1},i_{2},\ldots ,i_{N}}^{q}\;,$$
  where the phase space is divided into *n*-dimensional hypercubes of side *r* and $x_{i}=x\left(t=i\Delta t\right)$. The $p_{i_{1},i_{2},\ldots ,i_{N}}$ is the joint probability that the trajectory $x\left(t=\Delta t\right)$ is in the box $i_{1}$, x(2Δt) is in the box $i_{2}$, and $x\left(t=N\Delta t\right)$ is in the box $i_{N}$.
  The *Rényi entropy* is a special case of the *Kolmogorov-Sinai entropy*, i.e., for a finite time interval (NΔt=1), and for the probability that is not changing with the cell diameter ($m\left(r\right)=\mathrm{const}.,p_{i_{1},i_{2},\ldots ,i_{N}=p_{i}}$).

### 4.6. Shannon’s Entropy

Given a signal $\left[x_{1},x_{2},\ldots ,x_{n}\right]$ we then find the probability to occur for each value denoted as $P\left(x_{1}\right),\ldots ,P\left(x_{n}\right)$, thus we formulate Shannon’s entropy [50], as:

(18) $$H_{\mathrm{Shannon}}\;=\;-\sum_{i=1}^{n}P\left(x_{i}\right)\log_{2}P\left(x_{i}\right)\;.$$

giving units as bits, as the base of the logarithm is set to 2. Shannon’s entropy is a measure for the uncertainty of a (random) process/signal.

For related complexity measures, see Section 4.5.

Ref [27] uses Shannon’s entropy first to analyze the data under study and second as a tool for feature selection to improve predictions.

Ref [31] uses Shannon’s entropy as an additional feature in the input for the employed Machine Learning algorithms to improve predictions.

Shannon’s entropy is used in [33] to filter LSTM ensemble predictions and to discuss all data under study.

### 4.7. Wavelet Entropy

Following [51,52,53]: Wavelet analysis introduces an appropriate basis and characterization of a signal by a distribution of amplitudes in the bass. Furthermore, one would construct this basis as an orthogonal basis, which has the advantage that arbitrary functions can be uniquely decomposed and recomposed, i.e., the decomposition can be inverted.

A family of wavelet packets can be understood as elemental signals obtained from appropriate linear combinations of wavelets, i.e., the wavelets constitute the signal. Wavelets are locally-oscillating forms of sines and cosines, i.e., they have a good localization on both frequency and time.

We can find a wavelet family $\psi_{a,b}\left(t\right)$, where *t* is the time, as the set of elementary functions generated by a *mother wavelet*$\psi \left(t\right)$:

(19) $$\psi_{a,b}\left(t\right)={\left|a\right|}^{-\frac{1}{2}}\psi \left(\frac{t-b}{a}\right)\;.$$

Here a,b∈R and a≠0 are the scale/dilation and translation parameters, respectively.

We can then find two cases of wavelet transforms: The *continuous wavelet transform* (CWT) and the *discrete wavelet transform* (DWT). Both act on a signal $x\left(t\right)\in L^{2}\left(R\right)$ (the space of real square sumable functions).

The CWT of a continuous signal $x\left(t\right)$ is defined as the correlation between the function $x\left(t\right)$ and the wavelet family $\psi_{a,b}\left(t\right)$ for each *a* and *b*:

(20) $$T\left(a,b\right)=\frac{1}{\sqrt{a}}\int_{-\infty }^{\infty }dt\;x\left(t\right)\psi^{*}\left(\frac{t-b}{a}\right)\;,$$

where $\psi^{*}\left(t\right)$ is the complex conjugate of the analyzing wavelet function $\psi \left(t\right)$.

The DWT of a continuous signal $x\left(t\right)\in L^{2}\left(R\right)$ is found by first discretizing the wavelet family:

(21) $$\psi_{m,n}\left(t\right)=\frac{1}{\sqrt{a_{0}^{m}}}\psi \left(\frac{t-nb_{0}a_{0}^{m}}{a_{0}^{m}}\right)\;.$$

Here, contrary to the CWT, we find two integer parameters m,n which control the scale/dilation and translation, respectively. For the fixed dilation and location step parameters $a_{0}$ and $b_{0}$ we further find find the conditions $a_{0}>1$ and $b_{0}>0$. Here a common choice is $a_{0}=2$ and $b_{0}=1$, which is referred to as dyadic grid scaling. From now on we will use $\psi_{m,n}$ with the dyadic grid scaling as:

(22) $$\psi_{m,n}\left(t\right)=2^{-\frac{m}{2}}\psi \left(2^{-m}t-n\right)\;.$$

Whereas this constitutes an orthonormal basis of $L^{2}\left(R\right)$ of finite energy signals.

Next we assume the signal be given by sampled values, i.e., $x\left(t\right)=\left\{x_{1},x_{2},\ldots ,x_{M}\right\}$. If we now carry out the decomposition over all resolution levels, i.e., with $N=\log_{2}\left(M\right)$, we find the wavelet expansion as:

(23) $$x\left(t\right)=\sum_{m=-N}^{-1}\sum_{n}C_{m}\left(n\right)\psi_{m,n}\left(t\right)=\sum_{m=-N}^{-1}r_{m}\left(t\right)\;.$$

Here we interpret wavelet coefficients $C_{j}\left(k\right)$ as the local residual errors between successive signal approximations at scales *j* and j+1, and we thus call $r_{j}\left(t\right)$ the residual signal at scale *j*.

As the $\left\{\psi_{m,n}\left(t\right)\right\}$ are an orthonormal basis for the space $L^{2}\left(R\right)$ we find the concept energy derived the same way as in Fourier theory as:

(24) $$E_{m}={∥r_{m}∥}^{2}=\sum_{n}{\left|C_{m}\left(n\right)\right|}^{2}\;,$$

with the corresponding energy at each sample time step *n*:

(25) $$E\left(n\right)=\sum_{m=-N}^{-1}{\left|C_{m}\left(n\right)\right|}^{2}\;.$$

The total energy thus is:

(26) $$E_{\mathrm{tot}}=\sum_{m<0}\sum_{n}{\left|C_{m}\left(n\right)\right|}^{2}=\sum_{m<0}E_{m}\;.$$

We can then find the total wavelet entropy $S_{\mathrm{WT}}$ and the corresponding relative wavelet energy $p_{m}$ as:

(27) $$S_{\mathrm{WT}}≐S_{\mathrm{WT}}\left(p\right)=-\sum_{m<0}p_{m}\cdot \ln\left(p_{m}\right)\;,\mathrm{where}p_{m}=\frac{E_{m}}{E_{\mathrm{tot}}}\;.$$

Wavelet entropy is a measure of the order/disorder of a signal. Thus, it provides information about the corresponding dynamics and processes involved. This is because Shannon’s entropy idea, which for the wavelet entropy is used to analyze the different relative wavelet energies, gives a measure of the corresponding information of any distribution.

An ordered process can be interpreted as a periodic mono-frequency signal, thus having a narrow band spectrum. A wavelet representation of such a signal will almost entirely be resolved in one unique resolution level, i.e., all relative wavelet energies, but one will be almost zero. The non-zero one is thus the level that includes the representative signal frequency. For a signal like this, the corresponding wavelet entropy will be almost zero.

On the other hand, a random process would thus generate a very disordered signal. We expect a signal like this to have wavelet representations from all frequency bands. Further, one would expect these contributions to be of a similar order, much like the scaling seen in R/S analysis of a fractional Brownian motion. Thus, wavelet entropy will have its maximal values for signals like this.

In [30], wavelet entropy is used as an additional feature in the input for the employed algorithms to improve predictions.

### 4.8. Lyapunov Exponents of Time Series Data

The spectrum of Lyapunov exponents measures a system’s predictability depending on given initial conditions. For experimental time-series data, i.e., no different trajectories but only a single time series data, it still can be interpreted as a measure for predictability [54].

A Lyapunov exponent is a measure of exponential divergence, if, for a Lyapunov exponent λ, λ>0 and convergence, if λ<0, respectively. A system embedded in an *m*-dimensional phase space has *m* Lyapunov exponents. A higher value of a Lyapunov exponent corresponds to lower predictability, i.e., a higher degree of chaos. In general, a positive Lyapunov exponent indicates chaos [9], thus in most cases, it is enough to calculate the first Lyapunov exponent, i.e., the first and largest exponent of the whole spectrum. A system that has more than one positive Lyapunov exponent is referred to as *hyperchaotic* [55].

There is a range of algorithms/approaches to calculate the spectrum of Lyapunov exponents or just the largest Lyapunov exponent from a time series data, the interest reader thus is referred to [54,56,57,58,59,60,61]. However, discussing each of these approaches and their similarities/differences is not within the scope of this article. To get an idea on how to calculate Lyapunov exponents from time series, we discuss the algorithm by [57], as it is a practical and very understandable approach to estimate the largest Lyapunov exponent of a signal.

Given a signal $x_{i}=\left[x_{1},x_{2},\ldots ,x_{n}\right]$ we find a reconstructed phase space (We note that we excluded articles dealing with phase space reconstruction in particular, but, because we focus on the spectrum of Lyapunov exponents as a measure for signal complexity, we neglect this discrepancy.):

(28) $$X_{m}^{\tau }\left(i\right)\;=\;\left[x_{i},x_{i+\tau },\ldots ,x_{i+\left(m-1\right)*\tau }\right]\;,$$

with time delay τ and an embedding dimension *m*. The time delay can be found via the method of average mutual information from [62] and the *False Nearest Neighbours* algorithm [63] can be used to determine the embedding dimension.

The algorithm from [57] looks for the nearest neighbor of each point on the trajectory, the corresponding Euclidean distance $d\left(0\right)$ between two such neighboring points at instant 0 is defined as:

(29) $$d_{i}\left(0\right)=\min_{X_{m}^{\tau }\left(j\right)}d\left(X_{m}^{\tau }\left(j\right),X_{m}^{\tau }\left(i\right)\right)\;.$$

Thus, one finds the initial distance from the *j*th point to its nearest neighbor. Further, this algorithm is constrained, such that the nearest neighbors have a temporal separation which is greater than the mean period of the time series. The estimated largest Lyapunov exponent $\lambda_{1}$ then is the mean rate of separation of these nearest neighbors and is obtained from:

(30) $$d_{j}\left(i\right)\approx C_{j}e^{\lambda i\left(\Delta t\right)}\;,$$

with $C_{j}$ being the initial separation. Taking the logarithm thus yields:

(31) $$\ln\left(d_{j}\left(i\right)\right)=\ln\left(C_{j}\right)+\lambda_{1}i\left(\Delta t\right)\;,$$

where Δt is the sampling period of the time series. The corresponding distance $d_{j}\left(i\right)$ describes the distance between the *j*th pair of nearest neighbors after *i* discrete time steps. This can be interpreted as a set of approximately parallel lines, whereas the corresponding slope is roughly proportinal to the Largest Lyapunov Exponent $\lambda_{1}$. The largest LYapunov exponent is then estimated using a least-squares fit to an averaged line defined as:

(32) $$y\left(i\right)=\frac{1}{\Delta t}\langle \ln\left(d_{j}\left(i\right)\right)\rangle \;.$$

Here $\langle \cdot \rangle$ is the average over all values of *j*. This averaging allows an accurate evaluation of λ for short and noisy data.

In [7], the spectrum of Lyapunov exponents is used to analyze all the data under study, and the corresponding fractal interpolated data sets.

The spectrum of Lyapunov exponents is also used as filters for LSTM ensemble predictions in [33].

### 4.9. Fisher’s Information

As a measure of signal complexity, Fisher’s information is the amount of information extracted from a set of measurements, i.e., the quality of the measurements [64]. Another interpretation would be to use Fisher’s information to measure the order/disorder of a system or phenomenon. It is further well suited to investigate non-stationary and complex signals.

Given a signal $\left[x_{1},x_{2},\ldots ,x_{n}\right]$ we can find a discrete version of Fisher’s information.

Here, the first step is to construct embedding vectors:

(33) $$\vec{y}\left(i\right)\;=\;\left[x_{i},x_{i+\tau },\ldots ,x_{i+\left(d_{E}-1\right)*\tau }\right]\;,$$

with a fixed time delay τ and a corresponding embedding dimension $d_{E}$. The so constructed embedding space, as a matrix, is:

(34) $$Y\;=\;{\left[\vec{y}\left(1\right),\vec{y}\left(2\right),\ldots ,\vec{y}\left(N-\left(d_{E}-1\right)\tau \right)\right]}^{T}\;.$$

The next step then is to perform a single value decomposition on this matrix [65]. This yields a total of *Msingular values* σ. We can then find normalized singular values as:

(35) $${\bar{\sigma }}_{i}\;=\;\frac{\sigma_{i}}{\sum_{j=1}^{M}\sigma_{j}}\;.$$

Finally, Fisher’s Information then is:

(36) $$I_{\mathrm{Fisher}}\;=\;\sum_{i=1}^{M-1}\frac{{\left[{\bar{\sigma }}_{i+1}-{\bar{\sigma }}_{i}\right]}^{2}}{{\bar{\sigma }}_{i}}\;.$$

As given above, Fisher’s information requires two parameters, first the time delay, which can be found using the calculation of the average mutual information from [62] for the time delay and for the embedding dimension the *False Nearest Neighbours* algorithm [63] can be used (There are more methods to determine a correct phase space embedding, but as a phase space reconstruction is not the focus of this article we refer to the given ones as they are the most famous ones.). Thus, constructing a phase space embedding of the signal.

Fisher’s information is used in [33] to filter LSTM ensemble predictions and to discuss all data under study.

### 4.10. SVD Entropy

SVD entropy is an entropy measure based on a single value decomposition [65], of a corresponding embedding matrix, thus similar to Fisher’s information (Section 4.9).

It can be used to assess the predictability of stock market data, as done in [66,67].

To calculate SVD entropy, one constructs an embedding space for a signal $\left[x_{1},x_{2},\ldots ,x_{n}\right]$ with corresponding embedding vectors as [68]:

(37) $$\vec{y}\left(i\right)\;=\;\left[x_{i},x_{i+\tau },\ldots ,x_{i+\left(d_{E}-1\right)*\tau }\right]\;,$$

with a corresponding embedding dimension $d_{E}$ and time delay τ. The time delay can be found using the calculation of the average mutual information from [62] and the embedding dimension can be found by employing the *False Nearest Neighbours* algorithm [63] (There are more methods to determine a correct phase space embedding, but as a phase space reconstruction is not the focus of this article we refer to the given ones as they are the most famous ones.). Thus, constructing a phase space embedding of the signal, represented by a matrix:

(38) $$Y\;=\;{\left[\vec{y}\left(1\right),\vec{y}\left(2\right),\ldots ,\vec{y}\left(N-\left(d_{E}-1\right)\tau \right)\right]}^{T}\;.$$

The next step is then to perform a single value decomposition [65], on this matrix to get *M* singular values $\sigma_{1},\ldots ,\sigma_{M}$, i.e., the singular spectrum. A corresponding normalized spectrum of singular values is then found via:

(39) $${\bar{\sigma }}_{i}\;=\;\frac{\sigma_{i}}{\sum_{j=1}^{M}\sigma_{j}}\;.$$

And finally, using the concept of Shannon’s entropy (see Section 4.6) yields SVD entropy as:

(40) $$H_{\mathrm{SVD}}\;=\;\sum_{i=1}^{M}{\bar{\sigma }}_{i}\log_{2}{\bar{\sigma }}_{i}\;.$$

SVD entropy is used in [33] to filter LSTM ensemble predictions and to discuss all data under study.

### 4.11. Some Honorably Mentions for Measures of Signal Complexity

Furthermore, we want to give a list of other complexity measures, that from the authors’ point of view, make sense to be combined with machine learning approaches in one of the discussed ways (see Section 6).

- Generalized Hurst Exponent:
  The *generalized Hurst exponents* method is a tool to study the scaling properties of the data, and it is a generalization of the approach from Section 4.1. Note that the *generalized Hurst exponent* $H\left(q\right)$, contrary to the regular Hurst exponent, depends on a variable *q*, which means it is associated with the *q*th order moment of the distribution of the increments [69].
- Generalized Fractal Dimension:
  One can then find a connection between the Rényi entropy (see Section 4.5) and a *generalized fractal dimension* $D_{q}$. Following [48]:
  (41) $$S_{q}\left(r\right)\;=\;D_{q}\log_{b}r\;,$$
  where $S_{q}\left(r\right)$ is the *Rényi entropy* of order *q*, whereas q≥1 (for q→1 as a limit). $D_{q}$ is the corresponding generalized fractal dimension of order *q*, *b* is the base of the logarithm and *r* is the size of the cells of the embedding. For the generalized fractal dimension, we thus compute the probability with which the data points fall into the *i*-th cell.
- Approximate Entropy (ApEn):
  *Approximate entropy* was initially developed by Steve M. Pincus to analyze medical data [70] and afterwards adapted to general biologic network systems [71]. Later it spread it’s applications into various fields including finance [72] and psychology [73]. The motivation for this is that many approaches to calculate the entropy of a time series require huge data sets and cannot handle noisy data sets. *ApEn* assigns a non-negative number to a time series, where larger values indicate a greater randomness apparent in the process. From a statistical point of view, *ApEn* can be seen as an ensemble parameter of process auto-correlation, i.e., smaller values correspond to greater positive auto-correlation, larger values indicate greater independence.
- Sample Entropy (SampEn):
  Like Approximate entropy, sample entropy is a measure of complexity [72]. The difference to *ApEn* is that it does not include self-similar patterns and that it does not depend on the length of the data set.
- Range Entropy (RangeEn):
  The basic idea behind *ApEn* and *SampEn* is to calculate the Chebyshev distance between two state vectors in the reconstructed *m*-dimensional phase space [74]. However, this is limited because these formulations do not have upper limits.

## 5. Machine Learning Methods

In this section, we briefly discuss the used Machine Learning approaches. Note that, when we refer to machine learning approaches, we mean all sorts of data-based prediction/regression/classification algorithms that occurred in the listed research, such as k-nearest neighbors, Random Forest, Artificial Neural Networks, fuzzy Logic approaches, etc. Further, as there was a significant amount of papers featuring neural network approaches with varying architectures, we collected all of the neural network approaches together and again briefly discuss the employed architectures in the neural network context.

### 5.1. k Nearest Neighbours (*kNN*)

k nearest neighbours (kNN) [75] is a model-free algorithm for classification and regression. Like the name says, values or classes are predicted based on the *k* nearest neighbors, i.e., which class is dominant within these data points.

In [24], kNN, among other algorithms, is used to predict stock market data. Further, R/S analysis is used to identify non-random regions within the data.

### 5.2. Decision Trees

Decision trees are an algorithm for classification and regression that can be understood as that a tree-like structure to sort all occurring scenarios, thus containing only conditional control statements [76]. In [24], Decision Trees, among other algorithms, are used to predict stock market data. Further, R/S analysis is used to identify non-random regions within the data.

### 5.3. Random Forest

Random Forest [77], is an ensemble learning method for classification and regression analysis based on decision trees. The basic idea is to generate an ensemble of decision trees using bagging and later combine the results. The Idea of Bagging is to train many weak learners on the same data set and combine the result.

In [27], Random Forest, among other approaches, is used for electricity load forecasting. Furthermore, Shannon’s entropy is employed for feature selection.

Random Forest, among other algorithms, is used in [28] in conjunction with R/S analysis to predict stock market movement.

Two similar Random Forest algorithms are used in [17] to forecast different exchange rates. Furthermore, R/S analysis and the fractal dimension are used to analyze the data for predictability.

Random Forest, among other approaches, is used in [32] to predict the direction of US stocks. Here Effective Transfer entropy (ETE Section 4.4) is used as an additional feature to improve predictions.

### 5.4. Tree Based Extreme Gradient Boosting (XGBoost)

E**X**treme **G**radient **Boost**ing (XGBoost) is a tree based algorithm. The term *boosting* refers to combining the results of many weak predictions to a strong one, i.e., an ensemble of weak classifiers. Here, the selection of weak classifiers has to be optimized. Further, boosting is generalizable by allowing optimization of an arbitrary differentiable loss function.

XGboost was first proposed in [78] as part of greedy function approximations. Nowadays, the standard decision tree-based ensemble method was developed by [79].

In [32], XGBoost among other approaches was used together with Effective Transfer Entropy (ETE, see Section 4.4) to predict the direction of US stocks.

### 5.5. Logistic Regression

Logistic regression is a tool for regression analysis very similar to linear regression, but instead of a linear function, one employs a sigmoid function [80]. Logistic regression is useful for binary or multinomial dependent variables.

In [32], a Logistic Regression among other approaches was used together with Effective Transfer Entropy (ETE, see Section 4.4) to predict the direction of US stocks.

### 5.6. Support Vector Machines

The basic idea of Support Vector Regression [81,82] is to perform linear regression in a high-dimensional feature space using the kernel-trick, i.e., employing kernel functions. Thus, a linear model in the new space can represent a non-linear model in the original space. Support Vector machines and/or regression was first proposed in [83].

As previously mentioned, we map the input onto a high-dimensional feature space. Here one uses non-linear mappings to represent non-linear separations in the original space, i.e., one constructs an optimally separating hyperplane. The data points on each side of the hyperplane thus have a distance to the hyperplane. The smallest of these distances is called the margin of separation, and we will refer to the margin of the optimal hyperplane as *q* and the corresponding data points are called the *support vectors*. All other training examples are irrelevant for defining the class boundaries. SVM thus finds that particular linear model, i.e., the *maximum margin hyperplane*.

In [26], support vector machines are used together with a feature selection method based on the fractal dimension to predict the direction of stock indices on a daily basis.

Several approaches, among them, support vector machines, are used in [28] to predict stock market movement. Furthermore, R/S analysis is employed to test for market efficiency.

In [31], different forecast methodologies such as SVM are used to predict stock market behavior. Furthermore, Shannon’s entropy, Rényi entropy, and the Hurst exponent were used as additional features to improve the algorithm.

### 5.7. Multi-Linear Regression

Multilinear regression is the generalization of the standard linear regression model for multiple of dependent and independent variables [84,85]. It can be used to predict, e.g., prices. The basic idea is to find the correlation between two factors, i.e., the dependent and the independent variables. The corresponding assumption here is that the conditional mean function is linear.

In [31] mulit-linear regression and other approaches are used to predict stock market behavior. Furthermore, Shannon’s entropy, Rényi entropy, and the Hurst exponent were used as additional features to improve the forecasts. Ref [16] uses Multi-linear regression in addition to neural networks to predict the U.S./Mexico exchange rate. Furthermore, the fractal dimension of the time series data under study is used to analyze and improve the fuzzy logic approach.

### 5.8. Fuzzy Logic Learning Approaches

There exists a variety of fuzzy logic-based prediction methods, such as adaptive neuro-fuzzy inference systems (ANFIS [86]), Dynamic Evolving Neural-Fuzzy Inference System (DENFIS [87]), or Fuzzy Inductive Reasoning, (FIR [88]). All of these approaches can be broken down to the fuzzification of the data under study and learning rules, or train an algorithm, such as a neural network, based on these fuzzy sets, and then make predictions with the so learned behavior [89].

The work in [16] builds fuzzy rules based on both the actual time series data and the corresponding fractal dimension of the observed object to predict the data under study.

In [27], FIR, among other approaches, is used for electricity load forecasting. Furthermore, Shannon’s entropy is employed for feature selection.

Both ANFIS and DENFIS are used in [28] in conjunction with R/S analysis to predict stock market movement.

### 5.9. Artificial Neural Networks

One of today’s most common approaches to predict time series are artificial neural networks (ANNs) [90]. Artificial neural networks (sometimes just neural networks) are learning algorithms that emerged from the idea of human or animal brains. As such, they are constituted of connected neurons. Here, the way how they are connected is crucial for tasks they are employed for. Each neural network consists of an input layer, hidden layers, and an output layer. For our research, we categorize the mentioned applications into two types of neural networks. First, feed-forward neural networks, i.e., neural networks that are connected in a non-cyclic way, and recurrent neural networks, which are connected cyclically, i.e., the input and output or sub-parts of the network are connected such that the neurons form loops [91].

Further, the neurons have specific (non-linear) activation functions and are controlled by adjusting weights for each neuron. Thus, the learning process constitutes of adjusting and readjusting the weights on each neuron, through, e.g., backpropagation [92].

#### 5.9.1. Feed Forward Artificial Neural Networks

Feedforward neural networks are neural networks that are connected in a non-cyclic manner. The feed-forward neural network and its prototype, the multi-layer perceptron, were the first and most simple neural networks. Information propagates in only one direction, i.e., from input to output nodes.

Several different architectures for feed-forward artificial neural networks with backpropagation were tested in [19], such as with one hidden layer or two hidden layers, to predict stock market data. Here the Hurst exponent was used to indicate predictability of the time series under study, i.e., a long-term memory.

The study in [16] employs a three-layered feed-forward neural network with backpropagation to be tested against a combined fuzzy logic and fractal theory approach; further, the fractal analysis was applied to analyze all-time series that were to be predicted in this study.

In both [24,27] a standard three-layer feed-forward artificial neural network (one hidden layer) together with backpropagation was used to predict stock market data. In [24], the Hurst exponent was used to identify periods of time series that are easier to predict. Furthermore, in [27] Shannon’s entropy was used as a tool for feature selection.

Furthermore, in [31] a feed-forward artificial neural network with backpropagation was used. Again, the researchers employed a standard architecture of a three-layered ANN, thus one input layer, one output layer, and one hidden layer. Here, combinations of the Hurst exponent, Shannon entropy, and Rényi entropy were used as additional features to improve stock market predictions. In [30], again, a three-layered feed-forward artificial neural network was used to predict long-term trends of stock indices. Here wavelet entropy and the Hurst exponent were used as additional features for the algorithm to improve predictions.

Sometimes, or some types of feed-forward neural networks are referred to as Multi-Layer perceptrons. A multi-layer perceptron [90], refers to simple feed-forward artificial neural networks. The standard architecture consists of three layers, an input layer, a hidden layer, and an output layer. In contrast, the hidden and the output layer both consist of neurons with non-linear activation functions.

In [32], a MLP among other approaches was used together with Effective Transfer Entropy (ETE, see Section 4.4) to predict the direction of US stocks. In [23], an MLP is used together with the Hurst exponent to predict stock market data.

As it is very similar, we also discuss the cascade forward neural network in the context of feed-forward neural networks. The cascade forward neural network [93], has the same architecture as a feed-forward neural network but has a connection from the input and every previous layer to the following layers.

In [30], cascade forward neural networks are used together with R/S analysis and Wavelet entropy to improve forecasting for different time series data.

Another used architecture here is that of time-lagged neural networks. A time-lagged feed-forward neural network has a sliding time window as input. Thus, the input nodes (with the corresponding input) are ordered such that the input is a sequence. This architecture was used in [29] in combination with the Hurst exponent. Here, the Hurst exponent was used to identify time series data for better predictability and financial returns.

Though referred to as a time-delay neural network, which is usually used for speech recognition, the basic architecture of the neural network used in [21] is that of time-lagged neural networks. This research combines time-lagged neural networks with fractal analysis, e.g., Hurst exponent and fractal dimension, to predict stock price data. Here the fractal analysis improves the neural network architecture, i.e., tailor the input window.

Learning Vector Quantization (LVQ) is a supervised classification algorithm which can be used for classification and regression [94,95]. It is a precursor of Kohonen’s self-organizing maps [94], and is thus also inspired by the self-organizing capabilities of neurons in the visual cortex. It is a two-layered feed-forward neural network that uses a competitive (winner-take-all) learning strategy. In [30], (LVQ) was used, among other algorithms and by using the Hurst Exponent and wavelet entropy, to forecast financial time series data.

#### 5.9.2. Recurrent Artificial Neural Networks

Contrary to feed-forward neural networks are recurrent neural networks, where information can propagate in loops in the network. This architecture was specifically invented to learn temporal dynamic behavior, whereas speech recognition and time series prediction [96], are exemplary applications.

In [20], an artificial neural network with feedback connection, i.e., a recurrent neural network, was used to predict geomagnetic properties. Further, the local Hölder exponent was used as an additional feature to improve predictions.

In [23], an elmann recurrent neural network was used, among other architectures, together with the Hurst exponent, to tailor the input windows of the algorithms.

In [25], a recurrent neural network with embedded memory, i.e., a non-linear autoregressive model process with exogenous input (NARX), is used to forecast several different time-series data. Furthermore, R/S analysis, i.e., the Hurst exponent, is used to indicate and verify the predictability of the neural network approach.

Ref [28]: A Jordan neural network [97], was used among other approaches to predict stock market behavior. Furthermore, R/S analysis was employed to test for market efficiency.

A long short term memory (LSTM [98]) recurrent neural network, among other other approaches is used in [32] to predict the direction of US stocks. Here Effective Transfer entropy (ETE Section 4.4) is used as an additional feature to improve predictions.

In [7], a Hurst-exponent-based fractal interpolation is used to increase the data points of different time series data. These data are then forecast using a long short term memory (LSTM [98]) recurrent neural network. The fractal interpolation, and the forecasts are analyzed using a three different complexity measures, i.e., the fractal dimension (Section 4.2), the Hurst exponent (Section 4.1) and the spectrum of Lyapunov exponents (Section 4.8).

In [33], a Hurst-exponent-based fractal interpolation is used to increase the data points of different time series data. Then ensemble predictions are produced using randomly parameterized LSTM neural networks. Furthermore, finally, these predictions are filtered using five different complexity measures, i.e., the spectrum of Lyapunov exponents, The Hurst exponent, Shannon’s entropy, Fisher’s information, and SVD entropy. Further, this article uses a single hidden layer LSTM, gated recurrent unit (GRU [99]) and simple recurrent neural network as baseline predictions, i.e., to compare the ensemble results to.

## 6. Summary and Conclusions

This review analyzed the applications of a combination of complexity measures and machine learning algorithms for time series prediction. We found a total of 18 publications fitting our criteria (defined in Section 2).

Given the analysis discussed above, we found six types of combinations which we can sort all listed publications (Section 3) into (A table summing up these findings can be found in Appendix B):

1. Complexity measures as an additional criterion for analysis: [7,16,17,19,20,21,22,23,24,25,26,27,28,29,30,31,32,33]
2. Complexity measures to improve the architecture of the employed neural network/ algorithm: [21,23]
3. Complexity measures as additional features for machine learning algorithms: [16,20,30,31,32]
4. Complexity measures to find regions of increased predictability: [22,24,28,29]
5. Feature Selection using Complexity Measures: [26,27,29]
6. Filtering predictions/ensembles using Complexity Measures: [33]

The authors propose that combined approaches of machine learning and complexity measures have valuable contributions for both machine learning researchers and engineers as:

A complexity-based feature selection may save resources and improve the overall performance of the algorithm at hand.

An additional complexity feature can increase the accuracy of the algorithm at hand.

Analyzing the data and the predictions under study using complexity measures, one can argue for the quality of models based on their ability to recreate the complexity characteristics of the training data. Thus, the authors propose, complexity measures may become important for measuring the generalizability of models.

Models with higher accuracy and lower computational cost can be built taking into account the complexity of the data under study when designing the architecture of the algorithm, e.g., the neural network architecture.

Neural Network predictions can be sped up by circumventing the problem of finding the best set of hyper-parameters and instead choosingthe best predictions of different neural networks based on the complexity of the prediction.

Finding regions of increased predictability can yield higher returns for applications of machine learning in the finance sector.

Machine learning ensembles that are designed taking into account the complexity of the data under study may outperform today’s machine learning ensemble approaches.

## Data Availability

Not applicable.

## Appendix A. Measures of Signal Complexity: Summary Table

The following table summarizes the assertions, the applicability and the advantages/disadvantages of all discussed complexity measures from Section 4.

**Complexity Measure**	**Applicability**	**Assertion**	**Pros**	**Cons**
The Hurst Exponent	univariate time series data	persistency, anti-persistency, randomness	- only one parameter, i.e., the Hurst exponent - Hurst exponent lives within a fixed range, i.e., $H\in \left[0;1\right]$	- average over all scales, i.e., blind to multifractal behavior
Fractal Dimension of a Signal	univariate time series data	persistency, anti-persistency, randomness, sginal-complexity	- only one parameter, i.e., the fractal dimension - The fractal dimension of a signal lives within a fixed range, i.e., $d_{F}\in \left[1;2\right]$ - can be calculated from the Hurst exponent via $d_{F}=1-H$	- average over all scales, i.e., blind to multifractal behavior - if calculated from R/S analysis, it states the same as the Hurst exponent - many algorithms to choose from
The Local Hölder Exponent	univariate time series data	regularity, irregularity, varying scaling behavior	- can identify regions of different scaling behavior, i.e., multi-fractality	- varies from time step to time step - overall no comparable classification of time series data - yields one Hölder exponent for every time step
(Effective) Transfer Entropy	multivariate time series data	information transfer between time series data	- can detect non-linear Granger causal relationships - effective transfer entropy compensates for noise and non-stationarity	- only applicable between two time series data - transfer entropy contains noise - requires a large amount of data
Rényi Entropy	univariate time series data	diversity, uncertainty, randomness	- one can choose between weitghing events with higher or lower probability by adjusting a parameter *q*	- not recommended for real-valued data - requires and additional parameter *q*
Shannon’s Entropy	univariate time series data	diversity, uncertainty, randomness	- classical well understood complexity/information measure	- not recommended for real-valued data
The Spectrum of Lyapunov Exponents of time series data	univariate time series data	predictability, chaoticity,	- for many applications, it is sufficient to consider the largest Lyapunov exponent i.e., the first of the spectrum - the spectrum of Lyapunov exponents can be used to characterize the underlying dynamics of signals	- yields a spectrum of parameters - computationally expensive - only clearly defined for different trajectories and not for univariate data - for some algorithms a phase space embedding of the time series under study is required, i.e., the embdding dimension and the time delay
Fisher’s information	univariate time series data	quality of measurements, order/disorder	- well suited for non-stationary and complex signals - yields only parameter, i.e., the information content of the data under study	- requires two additional parameters, the embedding dimension and the time delay
SVD Entropy	univariate time series data	predictability, order/disorder	- well suited for non-stationary and complex signals - yields only parameter, i.e., the entropy of the data under study	- requires two additional parameters, the embedding dimension and the time delay
Wavelet Entropy	univariate time series data	order/disorder, randomness	- yields only one parameter, i.e., the wavelet entropy of a signal - provides information on underlying dynamics	- wavelet transform needs be performed beforehand - one has to choose a wavelet representation of a signal

## Appendix B. Results: Summary Table

The following table summarizes our findings. The entries of the fourth column are sorted using the different approaches featured in Section 6.

**Publication**	**Employed Complexity Measures**	**Employed Machine Learning Algorithms**	**Approach Type**
[19]	Hurst Exponent	Feed Forward Neural Network	1
[16]	Fractal Dimension	Feed Forward Neural Network Fuzzy Logic Approaches	1, 3
[20]	Local Hölder Exponent	Recurrent Neural Network	1, 3
[21]	Hurst Exponent, Fractal Dimension	Time Delayed Neural Network	1, 2
[22]	Hurst Exponent	Feed Forward Neural Network	1, 4
[23]	Hurst Exponent	Feed Forward Neural Network Recurrent Neural Network	1, 2
[24]	Hurst Exponent	Feed Forward Neural Network k-nearest Neighbours Decision Tree	1, 4
[25]	Hurst exponent	Nonlinear Autoregressive models with eXogenous input Recurrent Neural Networks	1
[26]	Fractal Dimension	Support Vector Machines	1,5
[27]	Shannon’s entropy	Fuzzy Inductive Reasoning Random Forest, Feed Forward Neural Network	1, 5
[28]	Hurst Exponent, Fractal Dimension	Adaptive Neuro-Fuzzy Inference System Dynamic Evolving Neuro-Fuzzy Inference System Recurrent Neural Network, Support Vector Regression Random Forest	1, 4
[17]	Hurst Exponent, Fractal Dimension	Random Forest	1
[29]	Hurst exponent, Fractal Dimension	Time Lagged Feed Forward Neural Network	1, 4, 5
[30]	Hurst Exponent, Wavelet Entropy	Feed Forward Neural Network Cascade Forward Neural Network Learning Vector Quanitzation	1, 3
[31]	Hurst Exponent Rényi entropy Shannon’s entropy	Multi-Linear Regression, Support Vector Regression Feed Forward Neural Networks	1, 3
[32]	Effective Transfer Entropy	Logistic Regression Feed Forward Neural Network (MLP) Random Forest, XGBoost Long Short Term Memory Neural Network	1, 3
[7]	Hurst Exponent The spectrum of Lyapunov exponents Fractal Dimension	Long Short Term Memory Recurrent Neural Network	1
[33]	Hurst Exponent The spectrum of Lyapunov exponents Fisher’s information SVD entropy Shannon’s entropy	Long Short Term Memory Recurrent Neural Network	1, 6
